# Newly Established Genetic System for Functional Analysis of MetSV

**DOI:** 10.3390/ijms241311163

**Published:** 2023-07-06

**Authors:** Finn O. Gehlert, Katrin Weidenbach, Brian Barüske, Daniela Hallack, Urska Repnik, Ruth A. Schmitz

**Affiliations:** 1 Institute for General Microbiology, Christian Albrechts University, 24118 Kiel, Germany; fgehlert@ifam.uni-kiel.de (F.O.G.); kweidenbach@ifam.uni-kiel.de (K.W.); bbarueske@ifam.uni-kiel.de (B.B.); dhallack@ifam.uni-kiel.de (D.H.); 2 Central Microscopy, Christian Albrechts University, 24118 Kiel, Germany; urepnik@bio.uni-kiel.de

**Keywords:** MetSV, *Methanosarcina*, archaea, genetic system, plasmid-born-virus, virus-host interaction

## Abstract

The linear chromosome of the *Methanosarcina* spherical virus with 10,567 bp exhibits 22 ORFs with mostly unknown functions. Annotation using common tools and databases predicted functions for a few genes like the type B DNA polymerase (MetSVORF07) or the small (MetSVORF15) and major (MetSVORF16) capsid proteins. For verification of assigned functions of additional ORFs, biochemical or genetic approaches were found to be essential. Consequently, we established a genetic system for MetSV by cloning its genome into the *E. coli* plasmid pCR-XL-2. Comparisons of candidate plasmids with the MetSV reference based on Nanopore sequencing revealed several mutations of yet unknown provenance with an impact on protein-coding sequences. Linear MetSV inserts were generated by BamHI restriction, purified and transformed in *Methanosarcina mazei* by an optimized liposome-mediated transformation protocol. Analysis of resulting MetSV virions by TEM imaging and infection experiments demonstrated no significant differences between plasmid-born viruses and native MetSV particles regarding their morphology or lytic behavior. The functionality of the genetic system was tested by the generation of a ΔMetSVORF09 mutant that was still infectious. Our genetic system of MetSV, the first functional system for a virus of methanoarchaea, now allows us to obtain deeper insights into MetSV protein functions and virus-host interactions.

## 1. Introduction

In contrast to many bacterial phage-host systems studied in recent decades, the number of isolated and described viruses in the archaeal domain is rather small but showed a higher diversity (reviewed in [1,2,3]). Dependent on suitable host systems, most described viruses infect archaea belonging to the *Halobacteria*, *Sulfolobales* or *Methanomicrobia* (reviewed in [4,5]). Although databases are growing daily, it is rarely possible to make accurate predictions about the function of genes of newly identified or isolated archaeal viruses since their genes show very few similarities to known genes [6]. In those cases, the generation of single gene mutations is an effective way to identify or verify gene functions. To perform genetic modifications, the genome must be accessible for manipulation. Today, very few genetic systems have been established for archaeal viruses. On the one hand, integrated proviruses can be directly modified in their hosts while in the lysogenic phase, as described by R. Selb and colleagues for the provirus ϕCh1 from *Natrialba magadii* [7]. Extrachromosomally present temperate viruses, which are present as plasmids like SNJ1 (pHH205) in *Natrinema* spp. strain J7-1, can also be genetically modified [8]. Moreover, anti-CRISPR- and CRISPR-based genome editing was described for *Sulfolobus islandicus* rod-shaped virus 2 (SIRSV2) [9]. However, for lytic viruses, it is usually unavoidable to modify their genomes in vitro and transform the resulting genomes into their respective host. In vitro transposon mutagenesis was described to be an efficient tool for the lytic haloarchaeal viruses SH1 and His2 to identify essential and non-essential regions of their genomes [10]. Further in vitro approaches based on the cloning of genomes into plasmids followed by site-directed mutagenesis (SDM) were described for various archaeal virus systems. The most studied genetic systems based on cloning the whole viral genomes belong to *Sulfolobus* infecting viruses, e.g., *Sulfolobus* spindle-shaped virus 1 (SSV1) or *Sulfolobus* turreted icosahedral virus (STIV) [6,11,12,13,14,15,16]. J. F. Wirth and colleagues showed a highly efficient viral genetic system for the characterization of STIV [6]. Generated plasmids consisting of the STIV genome in a pCRII-TOPO backbone were used to produce infectious virus particles by restriction of the resulting plasmid and spontaneous circularization of the viral insert, followed by transfection into *Sulfolobus* host strains [6]. Mutation of STIV open reading frames (ORFs) resulted in the identification of infection or replication-essential genes [6].

Although the knowledge of *Sulfolobus* infecting viruses might be larger to date, the ecological role of methanoarchaea is most likely even larger due to their methane production and its impact on the global carbon cycle and climate change (reviewed in [17,18,19,20]). On this basis, single organisms, as well as consortia, were of high research interest during the past sixty years regarding their biochemistry and physiology (e.g., [21,22,23]). The characterization of individual model organisms, e.g., *Methanosarcina mazei,* initially focused only on the biochemical characterization of methane production and regulation of nitrogen fixation [24,25,26,27], but also led to the isolation of viruses directed against methanoarchaea (reviewed in [1]). Consequently, nine viruses or virus-like particles have been described to infect methanoarchaea to date [1,28,29,30,31,32,33,34,35]. Within this small group of viruses, only two are infecting *Methanosarcina* species, *Methanosarcina* virus (MetMV) [29] and *Methanosarcina* spherical virus (MetSV) [35,36]. While MetMV was detected based on metagenomic approaches, MetSV was isolated from a waste-water treatment plant and can be propagated in the laboratory. MetSV, belonging to the *Tectiviridae*, infects *M. mazei* strains with a narrow host range [35,37]. When MetSV was first described in 2017, its linear genome of roughly 10 kb was predicted to encode 22 ORFs, all on one strand with the same direction of transcription and with a mostly unknown function [35,36]. In addition to the polymerase of type B, a potential ATPase and its capsid proteins, no functions could be assigned to the predicted proteins [35]. The genome was found to be flanked by imperfect terminal inverted repeats (TIRs; 5′-end: 5-GAGAGAGATAGGGTTGGGGATTCGCTTCGCTCACCCCAC-AACCCTGAGTAAGATTTTTG-3 (59 nct); 3′-end: 5-CAAAAATCTTACTCCGGGTTGCGGGGTGAGCGAAGCGAATCCCCAACCCTATCT-CGTCTTACTC-3 (64 nct)), but it has not been possible to determine whether the genome ends are sticky or blunt. Based on a Dual-RNAseq approach, interactions between host and virus proteins, as well as the role of host-encoded genes in the viral infection, were predicted but lacked proof-of-principle based on biochemistry or a genetic approach [36]. The analysis of total RNA isolated 30 or 180 min post-infection (p.i) suggested a potential network of small ORFs in combination with host-encoded genes, e.g., a single-strand DNA binding (SSB) protein potentially relevant for viral replication. Additionally, more general cell lysis with a key role of host genes was predicted and reduced the role of virus-encoded candidates for the host cell lysis [36]. Virus mutants are crucially important for the final verification of hypotheses based on those bioinformatic predictions.

Consequently, we report here on the establishment of a genetic system for MetSV to construct single gene knockouts to identify gene function. The PCR-amplified MetSV genome was cloned into the commercially available *E. coli* plasmid pCR-XL-2 as a baseline for genomic engineering of the virus (see Section 4). Linearized viral plasmid-inserts were able to infect *M. mazei* Gö1 with the production of viral particles as confirmed by transmission electron microscopy (TEM) and showed a comparable infection efficiency as the wildtype virus. Overall, the system we developed is the first functional genetic system for a methanoarcheal virus that allows us to construct gene knockouts of individual MetSV genes and elucidate their functions in combination with complementation experiments.

## 2. Results

### 2.1. Re-Position and Re-Annotation of the MetSV Genes

**Figure 1:**
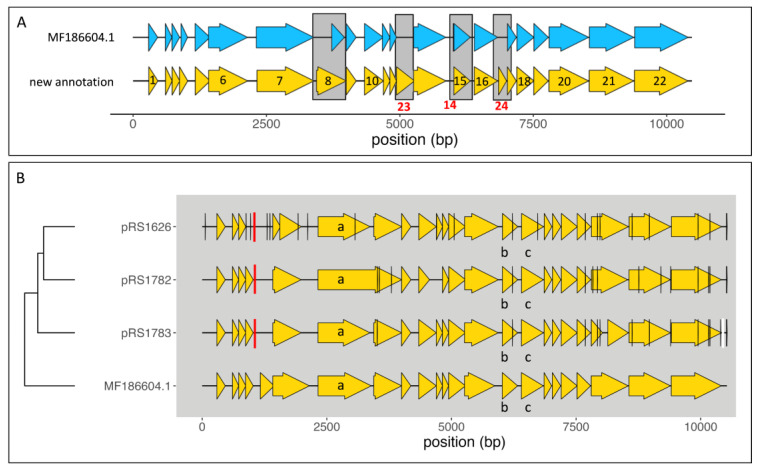
Sequence analysis of MetSV-derived plasmid inserts. (**A**) Visualization of the original MetSV genome annotation based on the NCBI database (https://www.ncbi.nlm.nih.gov/nuccore/1243285084 (accessed on 12 January 2023); blue) in comparison to the new annotation MetSV genome of the current work (yellow). New ORF IDs MetSVORF23 and MetSVORF24, as well as the missing one (MetSVORF14) are highlighted in red. (**B**) Clustering of the three cloned MetSV inserts named by their plasmid numbers pRS1626, pRS1782 and pRS1783, in comparison to the newly annotated MetSV genome. The depicted dendrogram was generated with *clustal omega* and visualized by *ggtree* and *ggplot2*. Viral ORFs were predicted using PROKKA in combination with PHROG database. Important genes are marked with small letters, e.g., a = type B DNA polymerase, b = small capsid protein and c = major capsid protein. Vertical lines indicate SNPs (black), insertions (red) and deletions (white).

Since the study aimed to select cloned MetSV genomes for the construction of a functional genetic system, the original MetSV reference (MF186604.1) was first re-annotated using PROKKA with a custom Hidden Markov Model (HMM) database based on the Prokaryotic Virus Remote Homologous Groups (PHROG) database (see Section 4). PROKKA predicted two new ORFs in previously non-coding regions. MetSVORF23 was predicted between MetSVORF12 and MetSVORF13, while MetSVORF24 was detected between MetSVORF16 and MetSVORF17 (Figure 1A; Table S1). MetSVORF23 showed an overlap with the sequence of MetSVORF12 due to sharing the last nucleotide of the stop codon of ORF12, which is simultaneously the first nucleotide of the start codon of the new ORF (5′-TG**A****TG**-3′). 

In addition to these newly detected ORFs, MetSVORF14 was no longer identified as an ORF on the new annotation (Figure 1A; Table S1). Further, MetSVORF04 was enlarged at the 5′-end of the coding sequences by 33 nucleotides (nct), while MetSVORF07 was shortened at its 5′-end by 12 nct (Figure 1A; Table S1). Additionally, the prediction and annotation of MetSVORF08 changed in general, its coding sequence was enlarged, but also the frame shifted by identifying the codon GTG as an alternative start codon (Figure 1; Table S1). These changes in the MetSVORF08 coding sequence result in a protein with 186 amino acids (aa) with a potential second polymerase function, in contrast to its previously predicted length of only 79 aa with an unknown function (Figure 1; Table S1). Both polymerase-encoding ORFs, MetSVORF07 and MetSVORF08, were found to be separated by 49 bp (Figure 1; Table S1).

### 2.2. Sequence Analysis of Cloned MetSV-Genomes

The MetSV genome was successfully PCR amplified using MetSV lysed *M. mazei* cultures as a template, cloned into the pCR-XL-2 vector and transformed in One Shot^TM^ OmniMAX^TM^ 2 T1*^R^* chemically competent *E. coli* cells as provided by the cloning kit (Thermo Fisher Scientific, Waltham, MA, USA; for details see Section 4). Three obtained candidate plasmids (pRS1626, pRS1782 and pRS1783) were isolated from selected single clones and analyzed by Oxford Nanopore Technologies (ONT; Oxford, UK) sequencing to verify correct viral inserts (Figure 1B). Plasmid inserts were assembled with the reference-based assembler *rebaler* (see Section 4, Figure 1B). To identify the best MetSV clone, assemblies were compared to the MetSV reference (MF186604.1) using multiple sequence alignments (MSAs) generated by *clustal omega* and were analyzed by *snippy* for single nucleotide polymorphisms (SNPs), insertions and deletions (INDELs). MetSV inserts were annotated by the same setup as for the reannotation of the reference using PHROGs (see Section 4, Figure 1B). The alignment, as well as the *snippy*-based analysis, gave an idea about SNPs and INDELs of the new assemblies in comparison to the used MetSV reference but further resulted in a clustering based on sequence identity. The *clustal omega* generated clustering gave the first evidence that pRS1783 showed a higher similarity to the NCBI reference than pRS1626 and pRS1782. The amount and location of SNPs and INDELs as well as their impact on coding sequences, were analyzed based on the detection with *snippy*. This analysis resulted in 14 to 22 variable positions per construct (Table S2). pRS1782 and pRS1783 inserts showed nearly equal SNP numbers. pRS1783 showed 14 SNPs, 1 insertion and 1 deletion, while pRS1782 showed 15 SNPs and 1 insertion (Figure 1B; Table S2). Differing from that, pRS1626 showed the highest number of variations represented by 21 SNPs and 1 insertion (Figure 1B; Table S2).

Roughly 34% of all detected variations (SNPs + insertions) were present in all three candidate MetSV inserts and consequently of the highest interest since these variations might be corrections of the originally published genome. C_10518_A and C_10525_T were introduced into the terminal inverted repeat (TIR) region at the 3′-end of the MetSV genome by the design of used primers in the PCR amplification prior to cloning since only one oligonucleotide was used matching both TIR regions, although both regions were differing by a few nucleotides. The role of the two introduced mutations in the non-coding regions of the 3′-end and the mutation represented by the SNP G_1049_GT in an intergenic region, which was present in most of all analyzed reads, seemed to be negligible (Figure 1A,C).

Variations in coding regions detected in most generated plasmids were T_6223_C, A_7685_G, G_7928_C, N_9945_T and T_10184_A. In four of five cases, these mutations led to changes in the encoded amino acids, with a potential impact on protein structures (Figure 1A,C). T_6223_C, located in the coding sequence of the small capsid protein (MetSVORF15), is the only silent point mutation among these detected SNPs, while A_7685_G led to an amino acid exchange of tyrosine to cysteine (Y56C) in the coding sequence of MetSVORF19, G_7928_C led to exchange R42P in the coding sequence of MetSVORF20 and the SNPs N_9945_T and T_10184_A, both located in the coding sequence of MetSVORF22 resulted in the amino acid exchanges V259D and I330M (Figure 1A,C). The discovered variation at nct 9945 may be a correction of the originally published MetSV genome, where it now seemed more likely to detect a T rather than another nucleotide.

### 2.3. Impact of SNPs and INDELs on Coding Sequences

**Figure 2:**
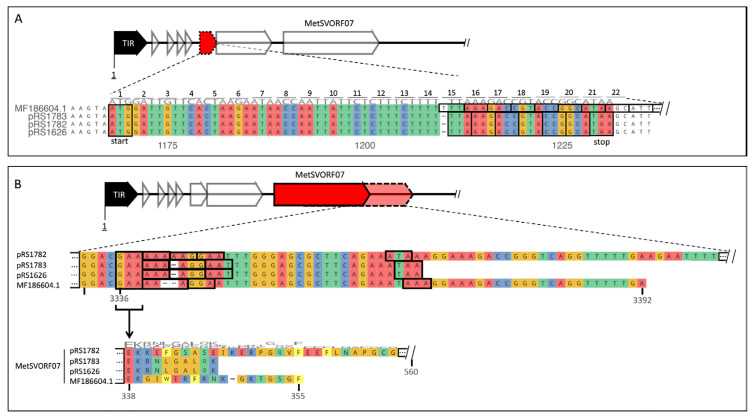
Alignments of the MetSVORF05 and MetSVORF07 region. (**A**) Deletion within a low complexity region led to frameshift mutation of MetSVORF05 (red) in the three cloned variants (pRS1626, pRS1782 and pRS1783), shortening the encoded peptide to 20 amino acids. This short ORF was not detected by PROKKA due to its detection limit for small ORFs below 30 aa [38]. (**B**) Nucleotide alignment of the 3′-end of the first polymerase subunit (red marked; MF186604.1; pRS1782; pRS1783; pRS1626). Highlighted is the region of the frameshift mutation (left black frames) and the resulting codon frames (right black frames). The corresponding amino acid alignment of the respective regions of the polymerase proteins is shown below.

MetSVORF05 encoded on the reference is not detected by PROKKA in the three plasmid-encoded versions due to a *snippy* undetected deletion in an AT-rich region, potentially causing a frameshift mutation. The frameshift mediated by TT_1210_T would result in an early stop codon after 60 nct representing the 20 amino acid peptide MDCSLRITNYSLSFLKTVPA* (Figure 2A). Due to the length cutoff of 90 nct of the gene prediction tool Prodigal as a part of the PROKKA pipeline, MetSVORF05 was not detected [38]. Further, many differences were found in MetSVORF07 encoding the viral polymerase of type B. Polymerase genes, encoded between nct 2325 and 3392, of the three variants were showing insertions of one or two As in the low complexity region between nct 3336 and 3344 (Figure 2B). These insertions were not detected by *snippy* and would have a huge impact on the encoded protein, especially in the case of pRS1782, which first polymerase gene would be massively enlarged to a final length of 1680 nct in comparison to only 1068 nct representing 355 amino acids of the reference (Figure 1C and Figure 2B). The potential changes in the polymerase of pRS1782 might be the reason why its viral insert was proven to be not infectious. Insertion of a single A in pRS1783 and pRS1626 resulted in a frameshift mutation, shortening the coding sequence by eight codons, while the insertion of two As detected in pRS1782 led to an enlarged encoded protein with 560 amino acids (Figure 1C and Figure 2B). In addition to these frameshifts, the SNP G_3065_C was detected in pRS1626 with an impact on the protein-coding sequence of the polymerase by amino acid exchange of K_251_N (Figure 1C). 

### 2.4. Transformation of Cloned Viral DNA Successfully Produced Virus Particles

**Figure 3:**
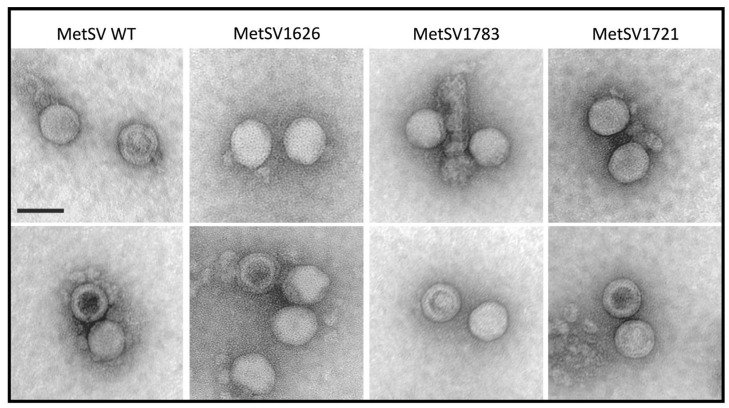
TEM imaging of plasmid-born MetSV particles. Completely lysed *M. mazei* cultures infected by the respective virus construct were filtered (0.022 µm), harvested by ultracentrifugation (>100,000× *g*; 4 h; 4 °C) and negatively stained. Intact, DNA-containing virions appear uniform, whereas empty capsids have an electron-dense center. Scalebar, 50 nm.

All cloned virus genomes were excised from their plasmids by BamHI restriction and used to transform *M. mazei* DSM3647 cells by liposome-mediated transformation (see Section 4). Host lysis was observed for transformation with the plasmid-born viruses MetSV1626, MetSV1783 and MetSV1721, which carried a deletion of MetSVORF09 (see below Section 2.5). Multiple transformations of MetSV1782 did not show virus particle production, potentially driven by the already shown changes in its polymerase; therefore, this construct was excluded from subsequent analyses. Plasmid-born virus particles produced by transformed *M. mazei* cultures were isolated from the supernatant by ultracentrifugation, negatively stained and visualized by TEM. Viral particles were observed for the two full MetSV insert plasmids, pRS1626 and pRS1783 (Figure 3). Additionally, to prove the principle of the genetic system, a complete deletion mutant of MetSVORF09 (pRS1721) derived from pRS1626 was developed by SDM. Transformation of the deletion mutant genome resulted in morphologically indistinguishable virus particles (MetSV1721) compared to the two generated constructs and the MetSV wildtype (Figure 3). 

### 2.5. Infection Studies of Plasmid-Born Viruses and Derivates

**Figure 4:**
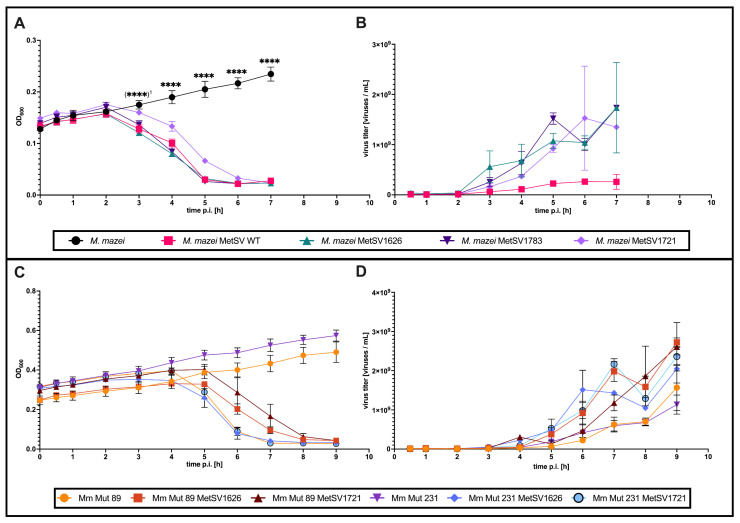
Infection studies of native MetSV and plasmid-born virions. Growth rates of infected *M. mazei* strains determined by OD_600_ measurements in combination with virus titers determined by absolute qPCR. (**A**) Depicted are OD_600_ values of three biological replicates of *M. mazei* wildtype (DSM 3647) infected with the different virus variants. Error bars indicate the standard deviation. Significant differences between the untreated controls and the virus-infected cultures based on two-way ANOVA with Tukey’s correction are indicated by stars. (****)^1^ highlights the time point 3 h post-infection (p.i.), where all infected populations first showed significantly lower densities compared to the untreated control (black), except for MetSV1721. (**B**) Corresponding development of virus titers over time determined by qPCR. (**C**) Complementation experiment of MetSVORF09 deletion mutant MetSV1721 by the overproduction of the viral gene in the *M. mazei* strain Mm Mut 231. As a control, the empty vector control strain Mm Mut 89 was treated similarly. (**D**) Corresponding virus titers of the complementation experiment shown in (**C**) determined by qPCR.

Lytic behavior—infection speed and viral offspring production—of plasmid-born viruses was characterized by challenging wildtype *M. mazei* cultures (DSM3647) with approximately 1.5 × 10^8^ viruses of each respective virus (MetSV WT, MetSV1626, MetSV1783 and MetSV1721 [ΔMetSVORF09]; Figure 4). The time course showed only small differences between the wildtype virus and the variants MetSV1626 and MetSV1783 (Figure 4A). Optical densities measured at 600 nm (OD_600_) of three biological replicates revealed similarities between the infection processes of *M. mazei* with the MetSV wildtype, MetSV1626 and MetSV1783 (Figure 4A). All variants led to the rapid and significant decline of the respective *M. mazei* populations starting 3 h p.i. (Figure 4A; (****)^1^). In contrast, MetSV1721 showed a slightly delayed lysis by approximately 1 h (Figure 4A). Interestingly, the virus titers determined for all three virus variants (MetSV1626; MetSV1783; MetSV1721) showed very similar values during growth analysis as well as at the endpoint of lysis, whereas the values for the wildtype MetSV were significantly lower (Figure 4B). In addition, growth analysis was performed using an *M. mazei* overproduction mutant of MetSVORF09 (Mm Mut 231) (Figure 4C,D). Here, the MetSVORF09 is encoded under the control of a trimethylamine (TMA)-inducible promoter on a shuttle vector (pSM02) [39]. The *M. mazei* strain Mm Mut 89—carrying an empty pSM02 plasmid—served as the empty vector control (Figure 4C,D). For the expression of MetSVORF09, TMA was added during the experiment (see Section 4). Growth was monitored by measuring optical densities. Again, cultures were infected with 1.5 × 10^8^ viruses MetSV1626 or MetSV1721 at an optical density of approximately 0.2 (Figure 4C). As expected, lysis by MetSV1721 occurred with a delay of approximately one hour in Mm Mut 89. The MetSV1721-infected overproduction mutant Mm Mut 231 lysed without delay and at the same rate compared with the non-mutant virus MetSV1626 (functionally complemented) (Figure 4C,D). In contrast to the observed differences in the cell lysis, the determined values of the virus titers were comparable across the different treatments (Figure 4D).

## 3. Discussion

For the generation of the genetic system, the MetSV genome was PCR amplified and cloned into a plasmid backbone. This followed a similar strategy to that described for the genetic system developed for the *Sulfolobus* virus STIV [6]. Three candidate clones were selected, sequenced and tested by transformation for their ability to produce infectious virus particles. The sequence analysis of the candidate plasmids was performed based on comparisons of long-reads to an updated annotation of the MetSV genome, as well as by reference-based assemblies and MSAs. The analysis led to the selection of the construct pRS1626 as the basis for the MetSV genetic system. The construct pRS1626, as well as a first generated MetSVORF09 deletion mutant of pRS1626 (pRS1721), were able to produce infectious virus particles in *M. mazei* transformations.

### 3.1. Updating the MetSV Annotation 

Since MetSV was described more than six years ago, databases and tools have been continuously updated; therefore, the original MetSV genome was reannotated with PROKKA with the addition of the Prokaryotic Virus Remote Homologous Groups (PHROGs) database (millard lab: https://millardlab.org/2021/11/ (accessed on 1 November 2022); [40]). The ORF prediction of Prodigal as part of PROKKA led to several changes in annotated MetSV ORFs and had some further impact on assigned gene functions in comparison to the original annotation performed with ORF Finder (http://www.ncbi.nlm.nih.gov/ (accessed on 2 March 2023); [35]). Prodigal has the clear advantage of using many different control parameters such as transcription start sites, codon usage, GC content, CDS overlap or similarities to known sequences in databases, whereas ORF finder is simply based on start and stop codons and a defined minimum length [38,41,42,43]. Therefore, ORF Finder often leads to false positives or highly overlapping CDSs [41], which for example, might be the case for MetSVORF14, as it overlapped with MetSVORF15 more than 87% in the original ORF prediction, and it was not identified in the new prediction. Further detected changes were derived by the prediction of a potential second polymerase subunit (MetSVORF08) and the new ORFs MetSVORF23 and MetSVORF24. However, PROKKA, in combination with PHROGs, was unable to predict functions for all MetSV genes in the reannotation, which shows the limits of the approach. One reason for this inefficient annotation of MetSV ORFs might be the underrepresented number of viruses belonging to the *Tectiviridae* in the PHROGs database [40]. Despite the herein presented update of the MetSV ORF prediction and potential functional assignments, these data are not contradictory to the proteomics data of previous publications [35]. However, additional genetic and biochemical experiments are highly essential to prove the correctness of the new ORF prediction and allow functional assignments.

### 3.2. Cloned MetSV Genome Construct as Basis for the Genetic System

Although the generation of genetic systems is a common and crucial technique in phage studies to unravel the function of unknown proteins, the higher complexity of the cultivation of archaeal hosts and their respective viruses might be the reason for a low number of archaeal systems [2,3,6,11,13]. Nevertheless, genetic systems are necessary, especially regarding MetSV, to gain deeper insights into the infection processes since bioinformatic predictions could assign functions to only a few MetSV genes, even with the PROKKA/PHROGs approach presented here [35,36]. Due to the lytic behavior of MetSV with its rapid host culture lysis induced within 4–5 h p.i. [35], combined with the less effective and time-consuming host cell transformation protocol [44], an in vivo genetic modification strategy was not suitable for MetSV. Instead, the ex vivo approach of cloning the entire MetSV genome into the pCR-XL-2 plasmid was chosen. This approach had the important advantage of not requiring selection markers, enabling all kinds of mutations from single nucleotides up to full gene deletions (reviewed in [2,6]). The generation of the system was facilitated by the small genome size of MetSV, as it allowed simple PCR amplification of the full MetSV genome by a long-amplification polymerase in a single fragment, followed by simple TA cloning. Consequently, complex PCR fragment assemblies of several PCR products with common ligase reactions or specialized techniques, e.g., Gibson assembly [2,6,45,46], were not required. For the previously mentioned cloning of STIV, three independent PCR products had to be assembled into a plasmid backbone since the STIV genome is about twice as large as the genome of MetSV [6,35]. The generated MetSV plasmid constructs, as well as their plasmid-born virus particles, were characterized by (i) sequence analyses regarding potential mutations, (ii) transmission electron microscopy as well as (iii) infection studies in a time course setting.

Sequence analysis of the three candidate constructs, pRS1626, pRS1782 and pRS1783, revealed multiple mutations based on the cloning procedure used and/or several years of virus propagation in the laboratory, with varying potential effects. Overall, 14 to 22 mutations per MetSV insert (≤2 × 10^−3^ substitutions per nucleotide) might represent the high mutation rates typical of viruses and phages during replication or could have arisen to some extent by PCR amplification during cloning [47,48,49]. In comparison to the archaeal example SIRV1, the number of detected mutations was very low, given the MetSV propagation in the laboratory for roughly six years. The determined mutation rate per replication cycle of SIRV1 was roughly estimated to be 3 × 10^−3^ substitutions per nucleotide per replication cycle [50]. This value seemed to be overestimated, as already mentioned by Prangishvili and colleagues since it would lead to roughly 30 SNPS/INDELs per generation in the MetSV genome. Although not all mutations might accumulate due to their potential lethal impact on the respective virus variant, the number of mutations per replication cycle of MetSV must be much lower and might significantly differ from mutation rates of the host’s genome due to the different used DNA polymerases responsible. In addition to the typical small mutations, no large-scale INDELs within the MetSV genome were detected. In contrast, large-scale deletions were described for archaeal viruses like ψM1 and SIRV1 [28,31,50]. The *Methanobacterium thermoautothrophicum* virus ψM1 was shown to spontaneously mutate during propagation in the laboratory to ψM2 by loss of a 0.7 kb fragment [28,31]. Further, SIRV1 was shown to spontaneously lose a 1.5 kb fragment with an impact on capsid size, which was found to be significantly smaller for the deletion mutant [50]. However, the sequencing data presented here could not completely exclude large INDELs because the *rebaler* assembler used cannot correctly represent such regions. Nevertheless, such changes are unlikely since the two infectious candidate constructs, pRS1626 and pRS1783, did not show obvious size differences in their capsids. However, potential SNPs/INDELs in coding regions would have a strong impact on gene expression, as predicted for the significantly elongated coding sequence of the polymerase of the pRS1782 insert, most likely resulting in an inactive enzyme and a non-viable virus. In case the alignment-based detection of a deletion in the T-rich region of MetSVORF05 is not an artifact due to potential sequencing or assembly error, this ORF would be significantly shorter, with only 20 amino acids left. In the case of SNPs/INDELs only detected by MSAs but not with *snippy*, these variations seemed likely to be such kind of errors as sequencing and assembly are more inaccurate in low complexity regions (LCRs) [51,52]).

Although the role and the correctness of the detected SNPs/INDELs have to be elucidated by further approaches, e.g., by genetics, biochemistry and Riboseq or proteomics, the constructs pRS1626 and pRS1783 were promising candidates as the origin for the MetSV genetic system, since their detected differences in coding sequences were less problematic than the detected uncertainty of the first polymerase subunit of pRS1782. Virus particles were only produced by the inserts of pRS1626 and pRS1783. 

The plasmid-born viruses MetSV1626 and MetSV1783 were showing equal lytic behavior in infection studies as the wildtype MetSV, although the cloned variants were indicating a trend to a more efficient reproduction based on higher final titers compared to the wildtype. This difference between plasmid-born viruses and the original wildtype virus might be the result of the introduced changes into the 3′-end of the MetSV genome during PCR amplification by using the shown PCR primer. This cloning strategy resulted in now fully identical chromosome ends with a potential impact on the viral replication, but further experiments are needed to validate this hypothesis. Furthermore, the role of the morphology of the chromosome ends must be investigated since the restriction procedure of the cloned plasmid constructs resulted in 5′-overhangs of four nucleotides, whereas the native chromosome ends of the wildtype MetSV remains unknown [35].

### 3.3. MetSVORF09 Function

As proof-of-principle of the generated genetic system, the constructed plasmid pRS1626 was used to generate a full MetSVORF09 knockout by SDM. MetSVORF09 was selected based on the sequence analysis of all the generated MetSV insert plasmids revealing no SNPs/INDELs in its coding sequence in any of the sequenced MetSV insert variants. This lack of mutations was also identified for MetSVORF01-03, 12, 15, 17, 18 and 24, with MetSVORF15 carrying only a silent point mutation (Table S3). Since a more stochastic distribution of small mutations was expected, as was described for SRIV1 [50], a more important role of the mutation-free gene subset was assumed. Deletions of these genes were generally expected to result in strong phenotypes like it was described for deletions of anti-defense genes [53] or replication essential genes like A197, C381 or B345 of STIV [6]. As confirmed by electron microscopy and infection analyses, MetSVORF09 was not essential for the MetSV infection, although its absence resulted in delayed host cell lysis. In trans complementation of the MetSVORF09 deletion by the *M. mazei* strain, Mm Mut 231 with a plasmid-born expression of MetSVORF09 resulted in host cell lysis by the MetSV1721 virus, which showed the same lysis dynamic as the non-mutated variant MetSV1626 again. The detected delay in the virus lysis, in combination with the detected similar virus titers, might indicate a role of MetSVORF09 in the virus particle release, while the deletion of MetSVORF09 did not affect the virus morphology. To obtain a better resolution of free virus particles and particles in infected but intact host cells, the qPCR-based quantification protocol needs to be optimized since the current virus titer determination method does not distinguish between free viruses and viruses in host cells prior to cell lysis.

Overall, we successfully established the first genetic system for a methanoarchaeal virus, allowing deeper insights into the MetSV infection process. Further, the ability to produce plasmid-born methanoarchaeal viruses might be of central interest in biotechnological applications, e.g., in the generation of new shuttle vectors or more efficient genetical tools.

## 4. Materials and Methods

### 4.1. Cloning of MetSV Genome into pCR-XL-2

**Figure 5:**
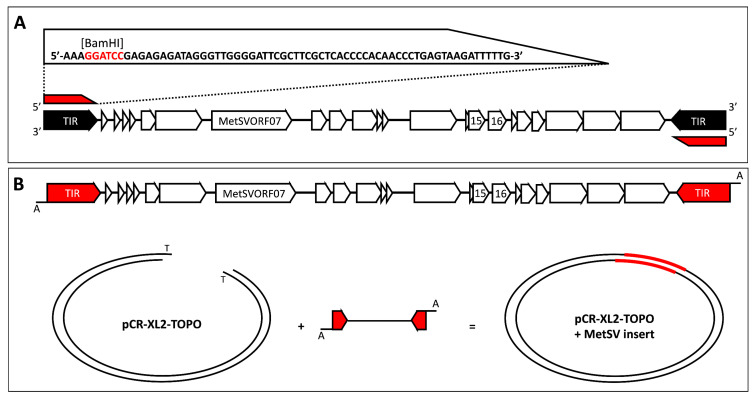
Summary of MetSV cloning procedure. (**A**) MetSV genome was amplified by PCR using the oligonucleotide MetSV_repeat_long (red) which was binding with a low number of mismatches to both MetSV genome ends (TIRs; black). (**B**) PCR product was ligated with TOPO vector according to manufacturer’s protocol.

MetSV genomes from a virus population were directly PCR amplified by an adjusted PCR protocol based on the Platinum SuperFi II proof-reading polymerase of the TOPO™ XL-2 Complete PCR Cloning Kit (Thermo Fisher Scientific, Waltham, MA, USA). Primer concentration of the used oligonucleotide MetSV_repeat_long (5-AAAGGATCCGAGAGAGATAGGGTTGGGGATTCGCTTCGCTCACCCCA-CAACCCTGAGTAGATTTTTG-3) of final PCR reaction was adjusted to 1 µM since both repeat areas at the 5′ and 3′ ends of the MetSV genome shared high similarity. The primer sequence covered the complete repeats and added a terminal BamHI cleavage site (Figure 5A). Infectious MetSV virions of *M. mazei* lysate were used as PCR templates. To support the long PCR reaction, especially in repeat regions, reactions were supplemented by 10 µL of optional GC Enhancer. After 35 amplification cycles according to the manufacturer’s protocol using an annealing temperature of 51.3 °C (10 s) and an elongation time of 6 min, the resulting PCR products were analyzed by gel electrophoresis (1% UltraPure™ Agarose, Thermo Fisher Scientific, Waltham, MA, USA). DNA fragments of roughly 10–11 kb were isolated from the gel after staining with ethidium bromide according to manufacture protocol of the PureLink Quick Gel Extraction and PCR Purification Combo Kit as part of TOPO™ XL-2 Complete PCR Cloning Kit, with the addition of 2-propanol in gel slice lysis. TOPO reactions, as well as following transformation of kit provided chemically competent One Shot^TM^ OmniMAX^TM^ 2 T1^R^ *E. coli* cells, were performed according to the manufacturer’s protocol (Figure 5B).

### 4.2. Single Clone Selection and Verification of Candidate Plasmids

Single clones were inoculated in 25 mL LB Medium containing 0.1 mM Kanamycin and incubated overnight at 37 °C and 200 rpm. Plasmid DNA was isolated using Presto™ Mini Plasmid Kit (Geneaid Biotech Ltd., New Taipei City, Taiwan). A total of 1 µg of plasmid DNA, isolated from overnight cultures of selected single clones, was restriction digested with BamHI-HF (New England Biolabs (NEB), Ipswich, MA, USA). Correct size of digestion products was verified by gel electrophoresis using MetSV genomic DNA isolated from lysed *M. mazei* cultures by ultracentrifugation at 126,100× *g* for 4 h at 4 °C followed by processing using QIAamp MinElute Virus Spin Kit (Qiagen, Hilden, Germany), according to manufacture protocol. Three candidate plasmids were selected (pRS1626, pRS1782 and pRS1783) and stored in *E. coli* glycerol cultures at −80 °C, named K4679, K4871 and K4872.

### 4.3. Sanger and Oxford Nanopore Sequencing

Plasmids pRS1626, pRS1782 and pRS1783 were sequenced by Oxford Nanopore sequencing on MinION platform using multiplexing via the Rapid Barcoding Kit SQK-RBK004 (Oxford Nanopore Technologies, Oxford, UK). Sequencing experiment was set up according to kit instructions for approx. 24 h. Basecalling and demultiplexing were performed using MinKNOW core software [v5.2.2] (Oxford Nanopore Technologies, Oxford, UK), including *guppy* [v6.2.7] (https://community.nanoporetech.com/downloads/ (accessed on 31 January 2022)) and *Bream* [v7.2.8]. FastQ passed reads were concatenated per barcode, quality filtered and trimmed using *LongQC* [v1.2.0c] ([54]; Supplementary File S1). Trimmed reads were length filtered by using *fastp* [v0.22.0] [55] at estimated maximum plasmid size (≤14 kb) based on gel electrophoresis as cutoff to reduce background noise and chimeras. 10 k of reads per barcode were selected randomly by using seqtk [v1.0-r82-dirty] (https://github.com/lh3/seqtk; accessed on 1 February 2022) and assembled with *rebaler* [v0.2.0] (https://github.com/rrwick/Rebaler; accessed on 1 February 2022) using MetSV reference MF186604.1. The final assemblies were manually curated by aligning the used primer sequence to the final assemblies. Genetic distances of inserts in comparison with MetSV reference were calculated using *clustal omega* [v1.2.3] [56,57] and visualized using R packages *ggplot2* [v3.4.0] [58], *gggenes* [v0.4.1] [59], *ggtree* [v3.6.2] [60,61,62,63], *treeio* [v1.23.0] [60,64] and *ggpubr* [v0.5.0] [65]. Nucleotide and protein alignments were visualized in R using ggmsa [v 1.2.3] [66]. Single nucleotide polymorphisms (SNPs), as well as INDELS (insertions and deletions), were analyzed using *snippy* [v4.6.0] [67]. Viral plasmid inserts were annotated using PROKKA [v1.14.6] [42] with PHROG database converted to hidden Markov models (HMMs) as reference database provided by the Millard lab (https://millardlab.org/2021/11/; (accessed on 1 November 2022)).

### 4.4. Growth of M. mazei and Generation of Mutant MetSV Lysates

*M. mazei* DSM3647 cultures were grown in 50 mL or 5 mL minimal medium supplemented with 150 mM methanol as described [35,44]. For liposome-mediated transformation, based on Ehlers and colleagues [44], 100 mL cells (OD600 > 0.6) were harvested by centrifugation for 10 min at 3000× *g* in oxygen-free atmosphere and carefully resuspended in modified sucrose buffer (0.15 M carbonate buffered sucrose 7.4 pH) by inverting. A total of 2 µg DNA—isolated viral DNA or BamHI-restricted plasmid DNA (pRS1626, pRS1782, pRS1783 and pRS1721)—was diluted in 50 µL sucrose buffer and added to mixture of 30 µL DOTAP (Roche, Basel, Switzerland) and 70 µL sucrose buffer. 990 µL of resuspended cells (>1 × 10^9^/mL) were added to DNA-liposome complexes after incubation for >30 min. Transformation reactions were incubated for roughly 5 h at 37 °C. Cells were split to ≈520 µL per transformation and transferred to 5 mL supplemented minimal medium containing an additional 0.1 M Sucrose. Cells were incubated overnight at 37 °C. 500 µL filtrated supernatant of lysed transformation cultures was transferred to 50 mL DSM3647 culture (OD_600_ ≈ 0.2) by filtration with 0.22 µm filters. Cell lysis was monitored by turbidity measurements. Reactions with identified cell lysis were used for verification by repeating lysis tests (≥3 biological replicates). Conformation of viral particle production was performed by TEM imaging of infected cultures. A total of 420 mL completely lysed cultures were filtrated through 0.22 µm filters (Sartorius, Göttingen, Germany) and harvested by ultracentrifugation at >100,000× *g* for 4 h at 4 °C. Pellets were carefully resuspended in water. TEM imaging was performed as described in [35].

### 4.5. Site Directed Mutagenesis (SDM) of MetSVORF09

MetSVORF09 knockout (ΔMetSVORF09) was generated by side-directed mutagenesis of pRS1626. pRS1626 was PCR amplified with the 5′-phosphorylated oligonucleotides [PHO]ATTTACATGTAAATTTACTTTTTTCTTATTTTTGTTTAAATCGATTCTCTTGATAT and [PHO]ATTTTCACCTCAATTAAATTTAGATTTAATTTTAGCTCTTCCAC by using the Platinum™ SuperFi II Green PCR Master Mix Kit (Thermo Fisher, Invitrogen, Waltham, MA, USA) like recommended. Resulting DNA fragments were DpnI treated at 37 °C for 1 h, followed by chloroform precipitation. Linear DNA fragments were circularized using T4 ligase (Thermo Fisher Scientific, Waltham, MA, USA) and transformed in chemical-competent *E. coli* DH5α following the method of Inoue and colleagues [68]. One kanamycin-resistant single clone was picked, and its plasmid was designated pRS1721. Respective MetSVORF09 deletion was verified by PCR and Sanger sequencing using MetSVORF8_for and MetSVORF10_rev. Plasmid-born MetSV1721 virus was produced as described above. 

### 4.6. Generation of M. mazei MetSVORF09 Overexpression Mutant

MetSVORF09 was initially cloned into the commercially available vector pET28a (NEB) using the primers MetSV_9_for_*NdeI* (5-CATATGATAAAACAAGACATTGAA-3) and MetSV_9_rev_*EcoRI* (5-GAATTCAAGAGAATCGATTTAAAC-3) for PCR amplification and the *NdeI* and *EcoRI* sites, which were inserted by the primers. The resulting plasmid was designated pRS1394. For cloning into pSM02 [39], MetSVORF09 was amplified using the primers pET28a-pSM02_*NheI*_for (5-GCTAGCGGATAACAATTCCCCTC-3) and pSM02-pET28a_*ApaI*_rev (5-GGGCCCCAAGCTTGTCGAC-3) and pRS1394 as template. The PCR product was cloned into pSM02 via the *NheI* and *ApaI* sites, resulting in plasmid pRS1421. pRS1421 was used to transform *M. mazei* wildtype cells (DSM3647) as described by Ehlers and colleagues [44], generating *M. mazei* mutant strain 231 (Mm Mut 231) (pRS1421). Furthermore, to generate an empty vector control *M. mazei* was transformed with pSM02 [39] to generate Mm Mut 89 according to the same protocol [44].

### 4.7. Infection Study of Plasmid Originated MetSV Variants

*M. mazei* wildtype (DSM3647) was grown in 50 mL *M. mazei* complex media using methanol as sole and carbon source as described [69]. For complementation analyses of the MetSVORF09-deletion, the *M. mazei* strain Mm Mut 231, providing MetSVORF09 under trimethylamine (TMA) inducible promotor (p1687 promotor) and Mm Mut 89 were grown in 50 mL *M. mazei* complex medium additionally supplemented with 20 µg/mL neomycin and 25 mM TMA. In early exponential phase at OD_600_ ≈ 0.2, a total amount of 1.5 × 10^8^ viruses MetSV (wt) or plasmid-born MetSV1626, MetSV1783, MetSV1721 was added to the respective number of biological replicates. Cultures were monitored by measurement of the OD_600_ and virus titers. Virus titer was determined with an adapted absolute qPCR protocol based on [36]. Prior to qPCR measurements, 10 µL of culture subsamples were treated with RNase A and DNase1 (both Thermo Fisher Scientific, Waltham, MA, USA) in a final volume of 100 µL for 1 h at 37 °C, followed by heat inactivation and denaturation of the intact virus capsids at 95 °C for 30 min. 1 µL of processed samples were used as templates for absolute qPCRs in comparison to the normalization plasmid pRS1332 carrying the coding sequence of the small capsid protein (MetSVORF15) in 10 µL qPCR reactions (PowerUp SYBR Green Master Mix; Thermo Fisher Scientific, Waltham, MA, USA). Please notice that consistent results required frequent vortexing during pipetting. The number of viral genomes, which in this context corresponds to the virus titer, was calculated like the absolute transcript numbers using the formula presented in a previous publication based on the same number of biological and technical replicates [36]. 

## Data Availability

Sequencing data of the generated MetSV insert variants (MetSV1626, MetSV1782 and MetsV1783) is provided under the following BioProject ID: PRJNA980962.

## Supplementary Materials

The supporting information can be downloaded at: https://www.mdpi.com/article/10.3390/ijms241311163/s1.
