# Supplementary material for: Newly Established Genetic System for Functional Analysis of MetSV
Source: Int J Mol Sci. 2023 Jul 6;24(13):11163. doi: 10.3390/ijms241311163 (PMC10342651; doi:10.3390/ijms241311163)

QC Summary pRS1626

General Statistics

General statistics

**Sample name**	-
**Yield**	171893067
**Number of reads**	32770
**Q7 bases**	85.604%
**Longest read**	44116
**Estimated non-sense read fraction**	0.129

Adapter Statistics

Adapter statistics

**Number of trimmed reads in 5'**	29913
**Max seq identity for the adpter in 5'**	1.000
**Average trimmed length in 5'**	110.559

Read length

Read length

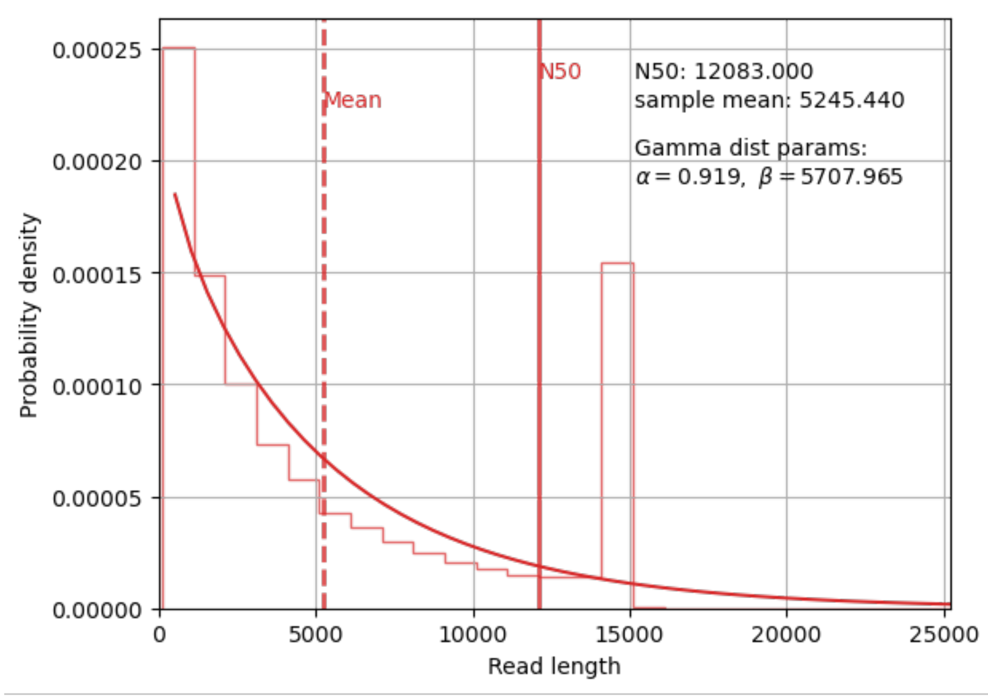

**Mean read length**	5245.440
**N50**	12083.000

Per Read Quality

Per read QV

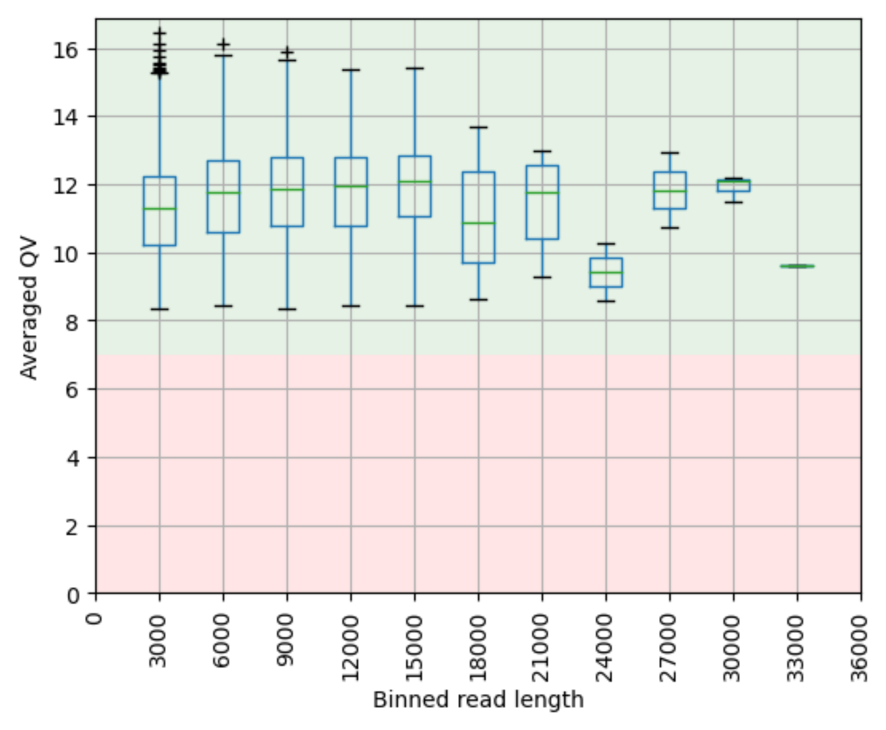

Per Read Coverage

Per read coverage

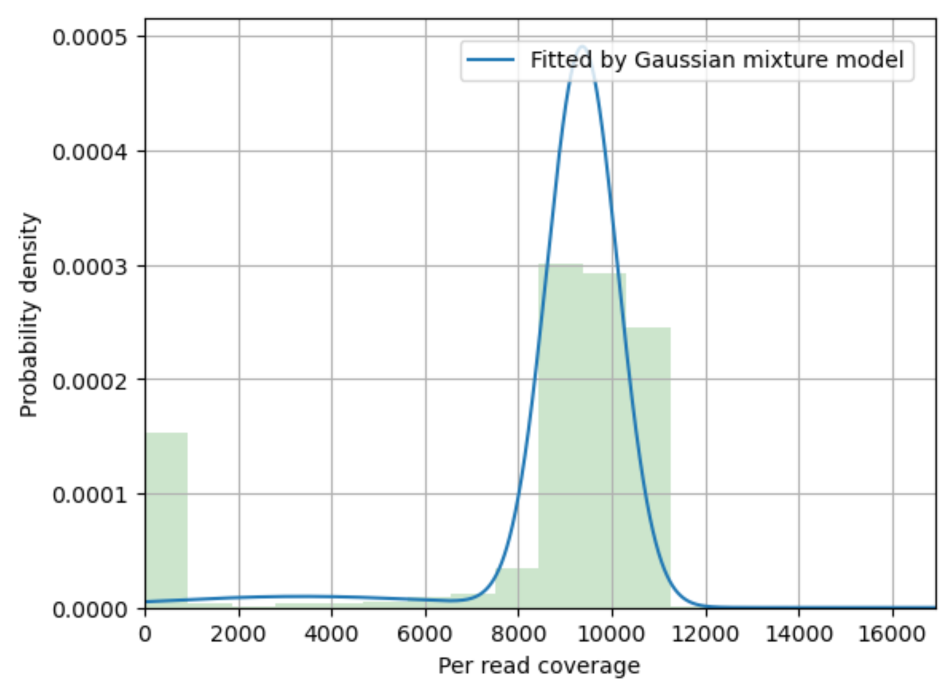

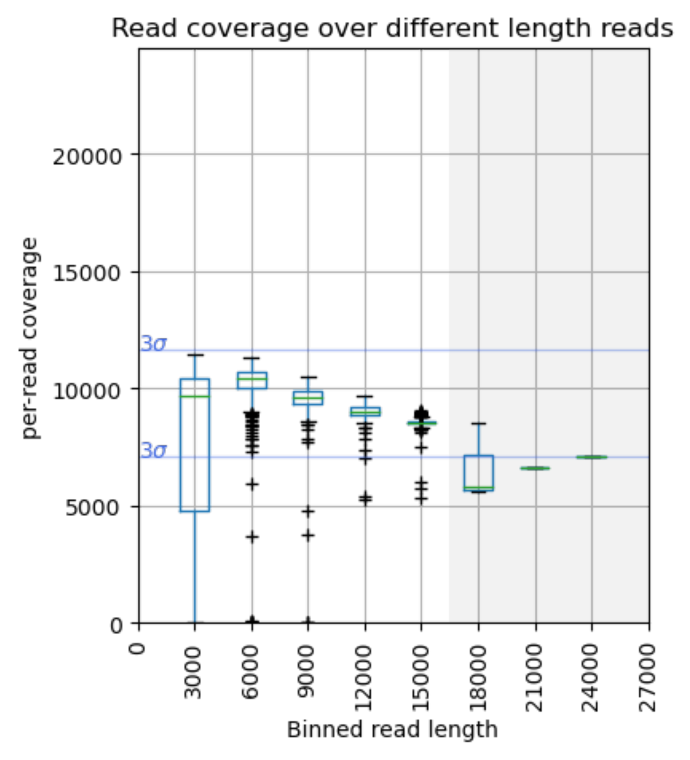

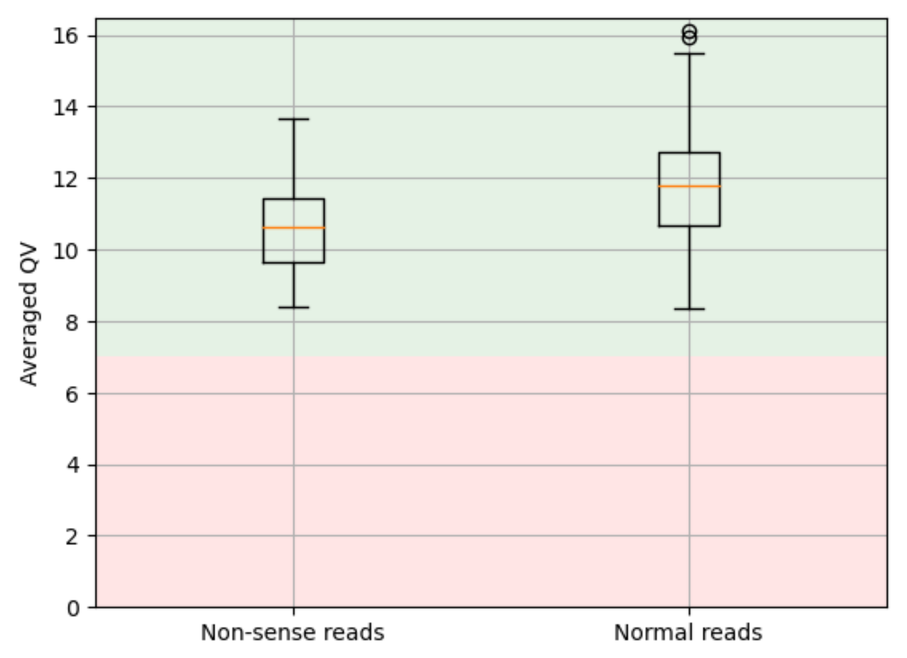

**Number of sampled reads**	5000
**Mean per read coverage**	9373.443
**S.D. per read coverage**	755.859
**Crude estimated Xome size**	15968 (e = 12.9%)

GC contents

GC contents

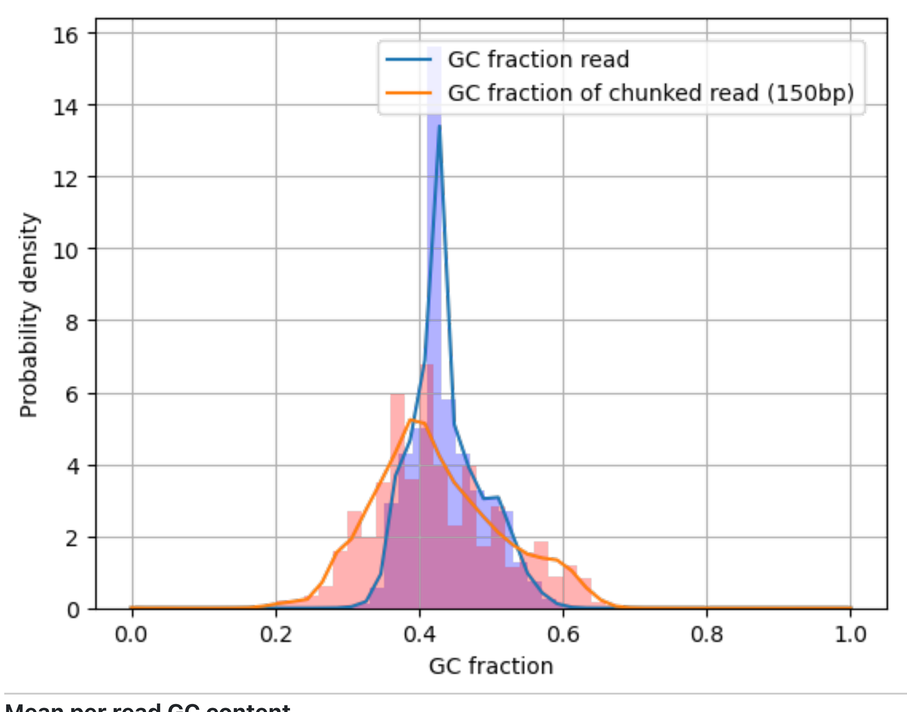

**Mean per read GC content**	43.719 %
**s.d. per read GC content**	4.875 %

Flanking region analysis

Flanking region analysis

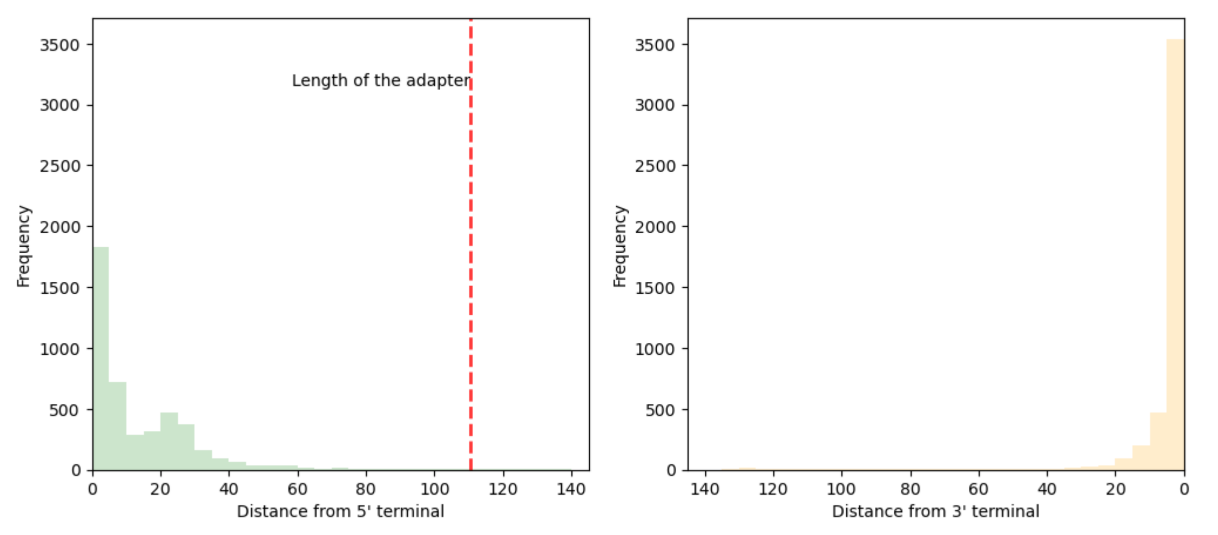

Sequence complexity

Sequence complexity

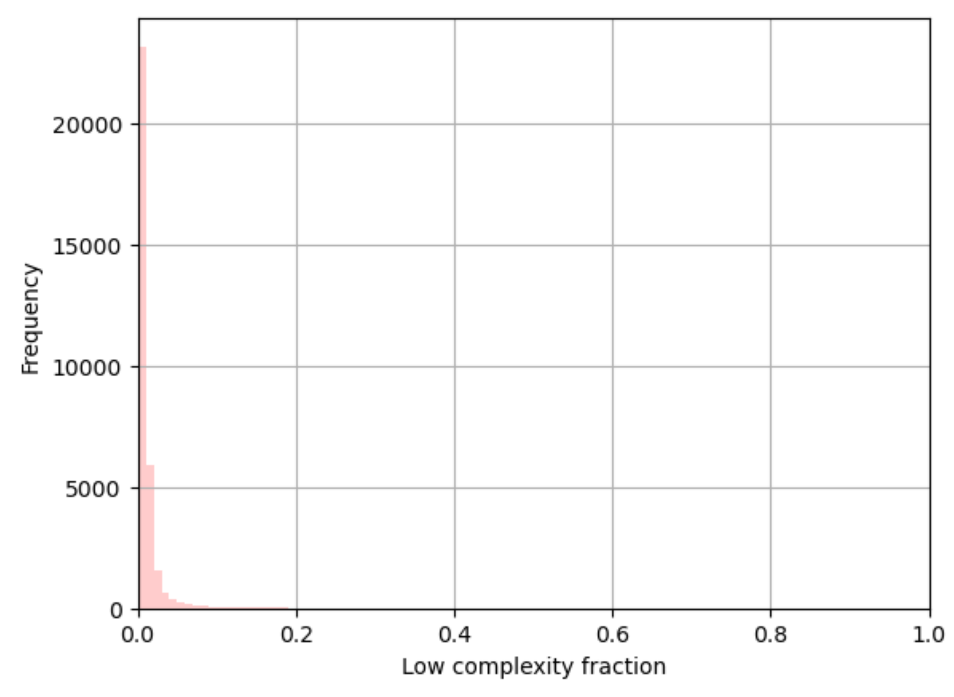

QC Summary pRS1782

General Statistics

General statistics

?

Sample name	-
Yield	187120918
Number of reads	26669
Q7 bases	85.691%
Longest read	28893
Estimated non-sense read fraction	0.139

Adapter Statistics

Adapter statistics

?

Number of trimmed reads in 5'	23962
Max seq identity for the adpter in 5'	1.000
Average trimmed length in 5'	111.215

Read length

Read length

?

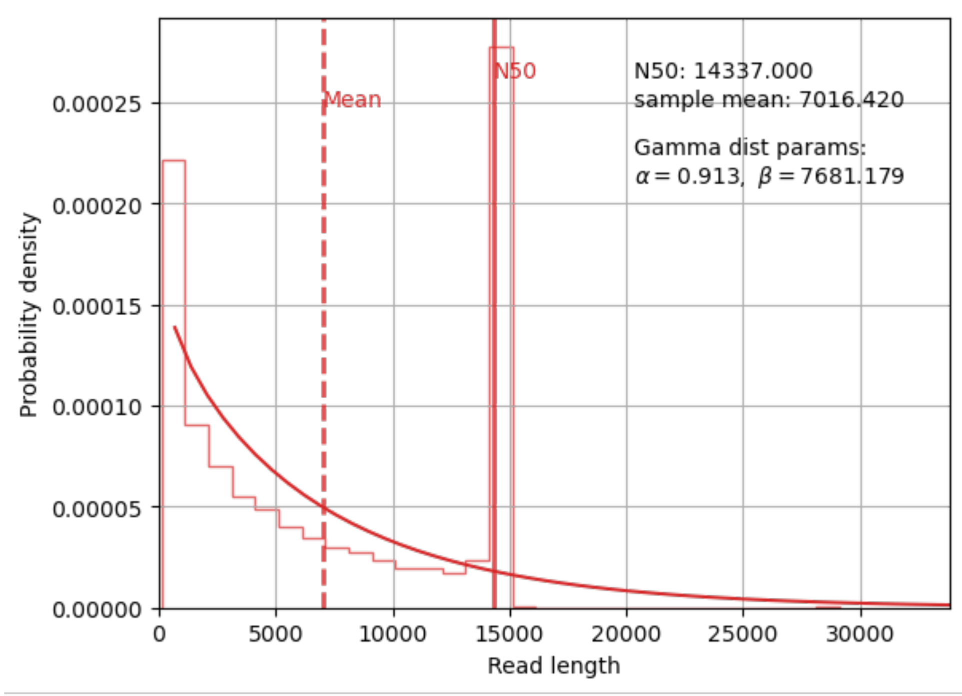

Mean read length	7016.420
N50	14337.000

Per Read Quality

Per read QV

?

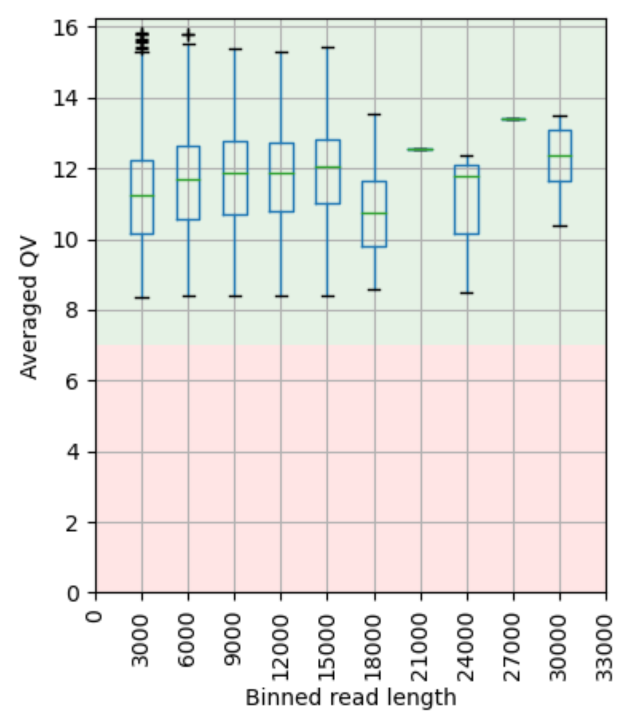

Per Read Coverage

Per read coverage

?

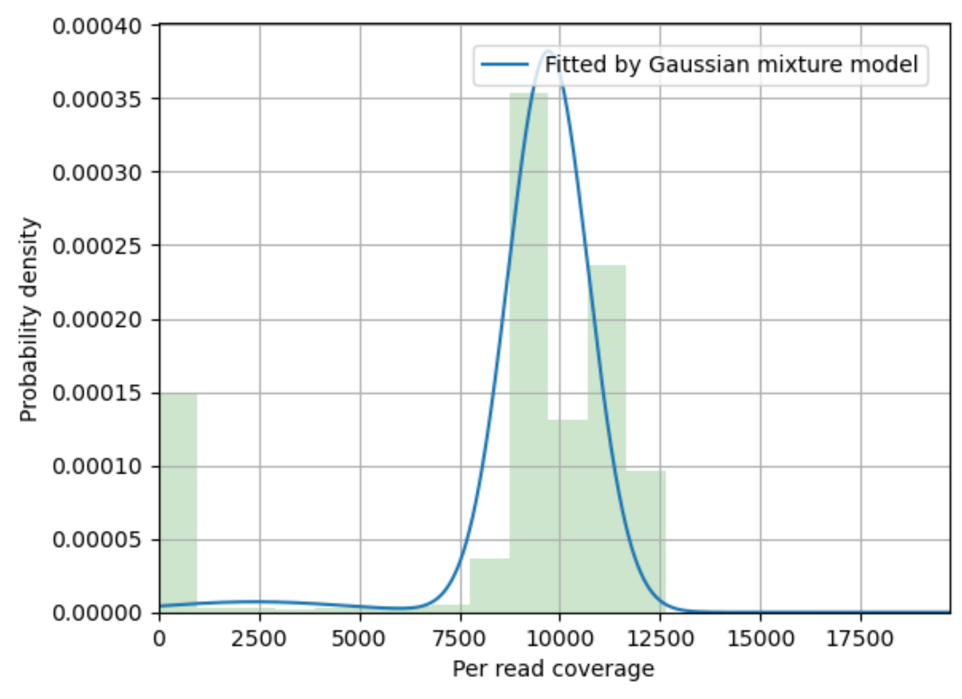

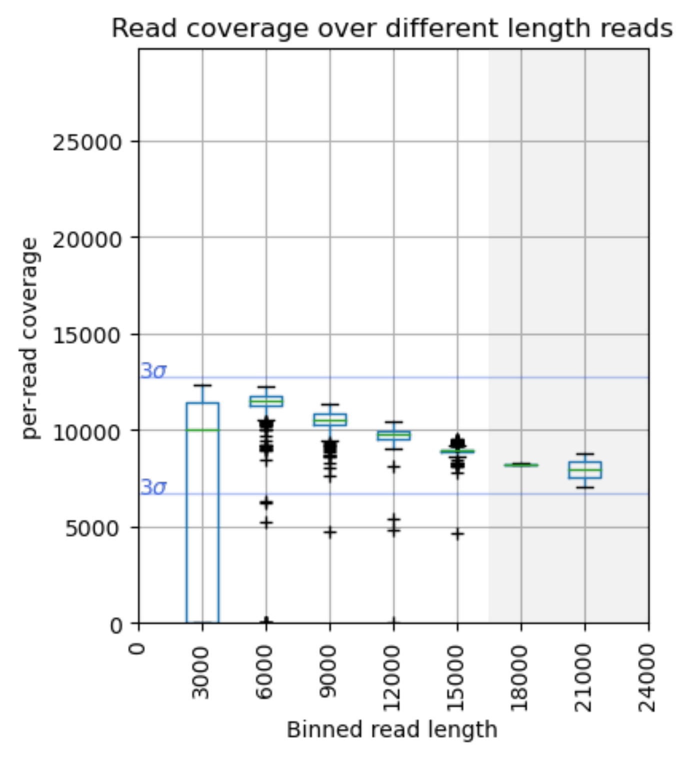

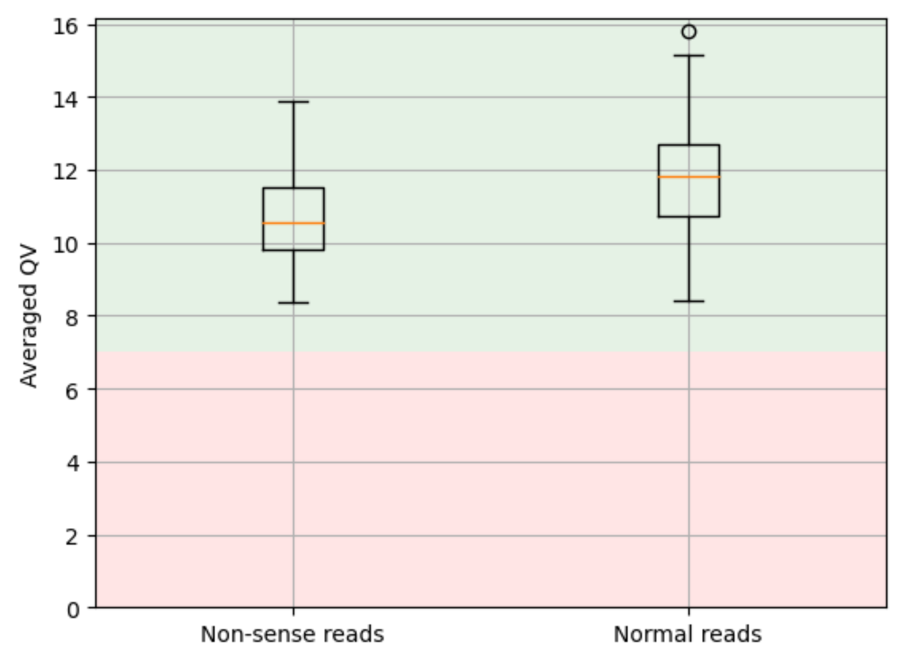

Number of sampled reads	5000
Mean per read coverage	9716.482
S.D. per read coverage	1000.327
Crude estimated Xome size	16577 (e = 13.9%)

GC contents

GC contents

?

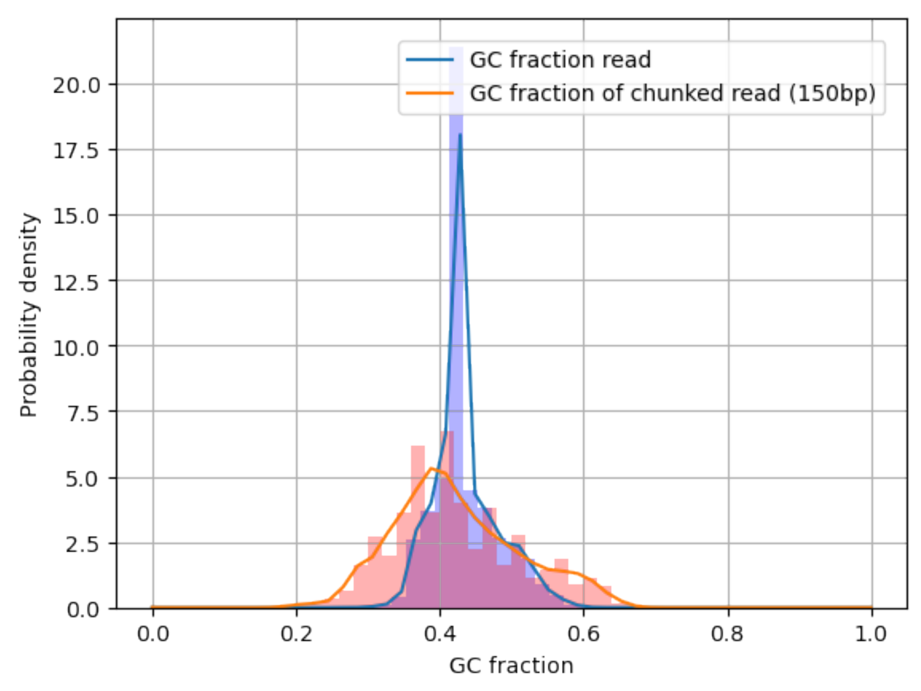

Mean per read GC content	43.397 %
s.d. per read GC content	4.354 %

Flanking region analysis

Flanking region analysis

?

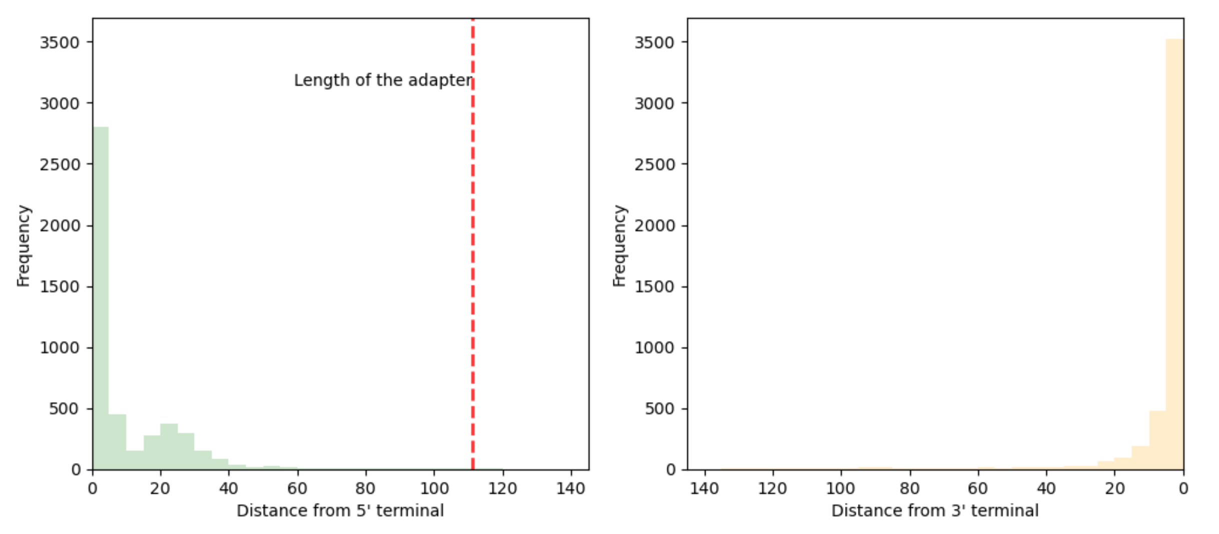

Sequence complexity

Sequence complexity

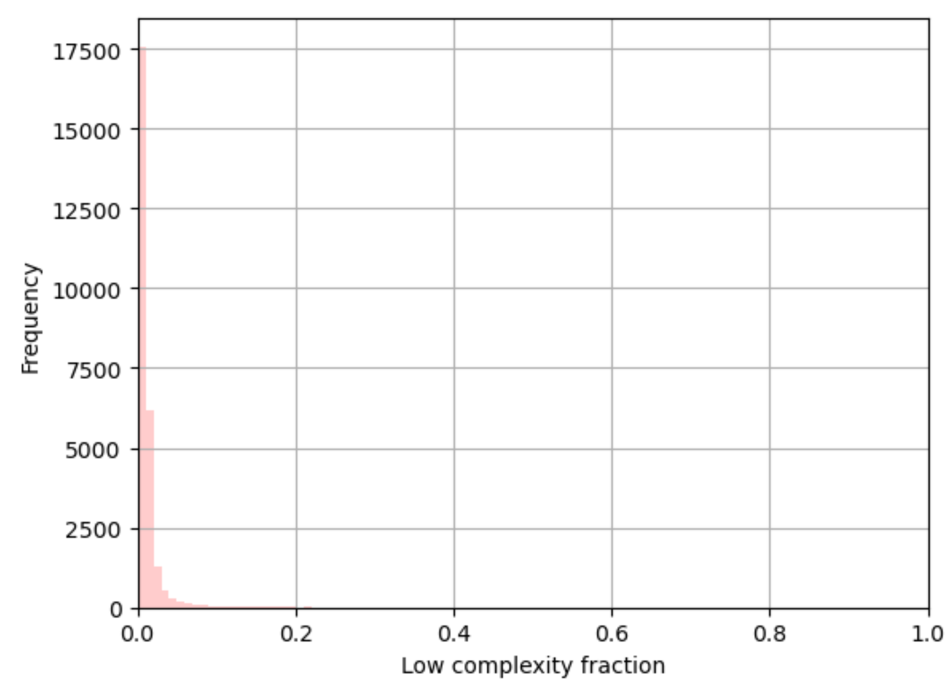

QC Summary pRS1783

General Statistics

General statistics

Sample name	-
Yield	372774915
Number of reads	50660
Q7 bases	85.391%
Longest read	53346
Estimated non-sense read fraction	0.180

Adapter Statistics

Adapter statistics

Number of trimmed reads in 5'	45006
Max seq identity for the adpter in 5'	1.000
Average trimmed length in 5'	111.169

Read length

Read length

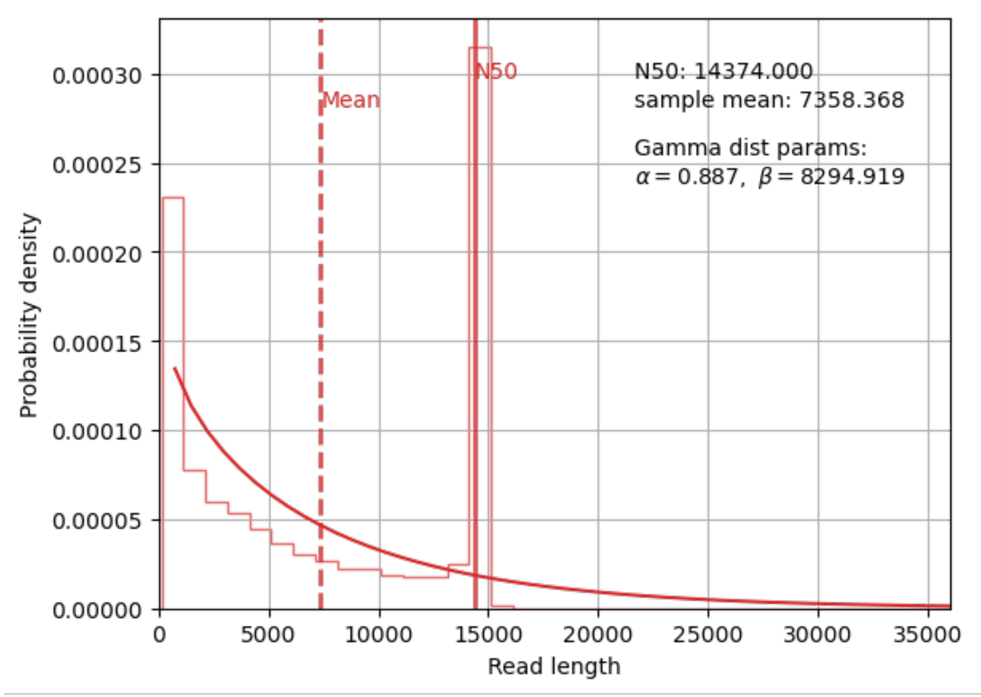

Mean read length	7358.368
N50	14374.000

Per Read Quality

Per read QV

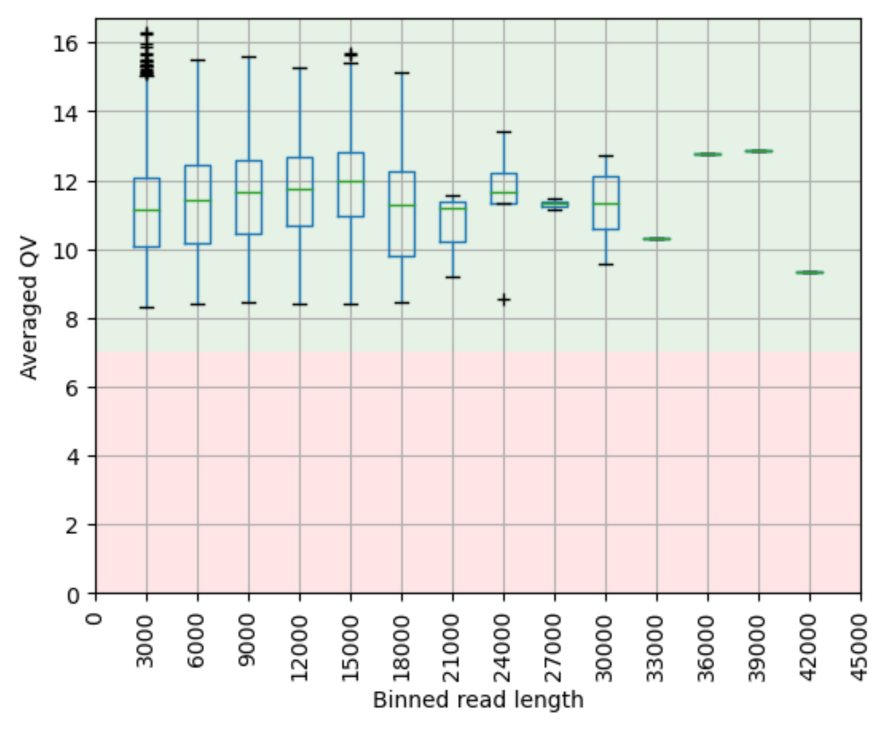

Per Read Coverage

Per read coverage

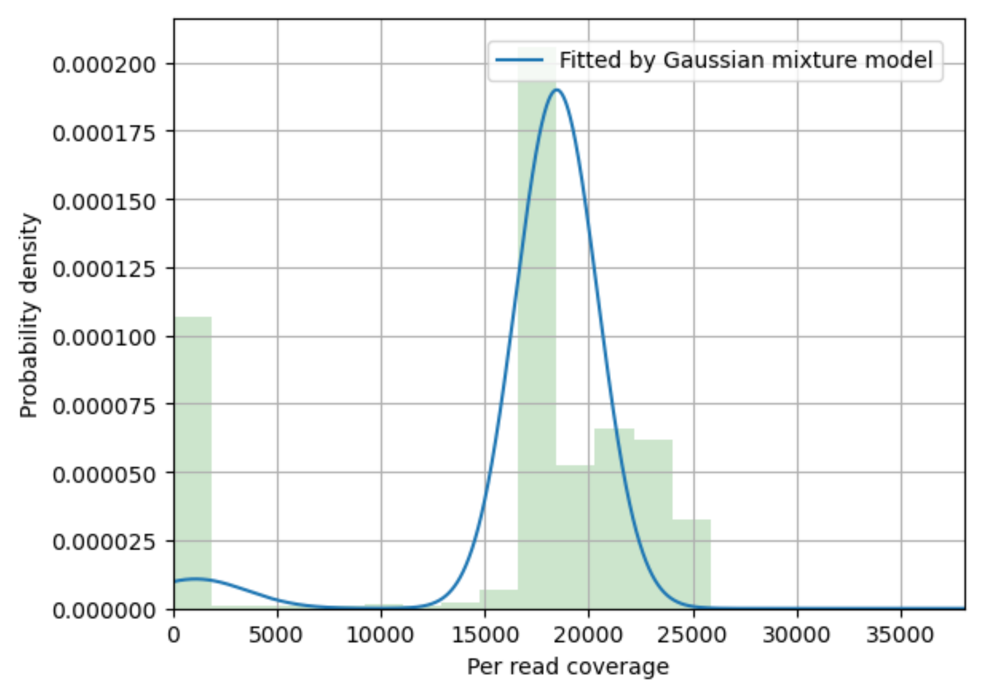

Read coverage over different length reads

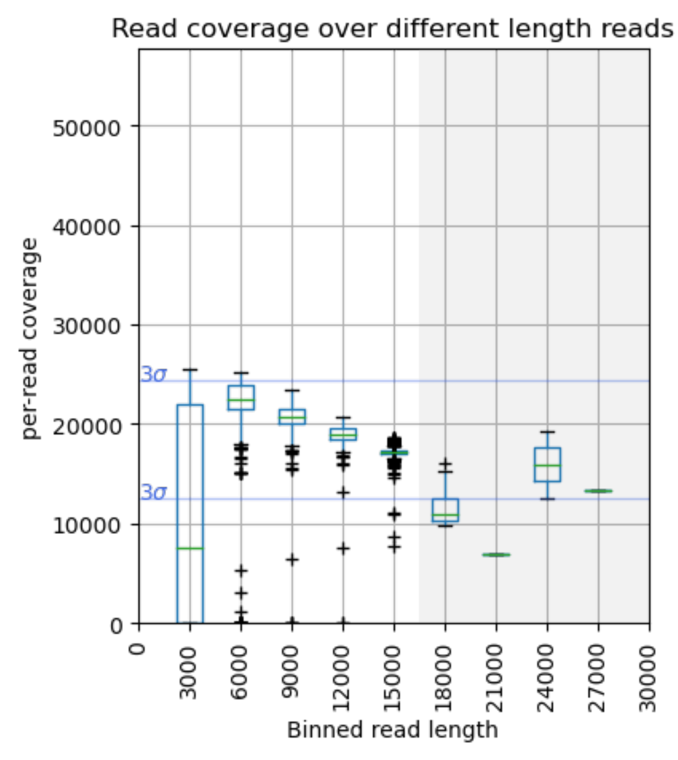

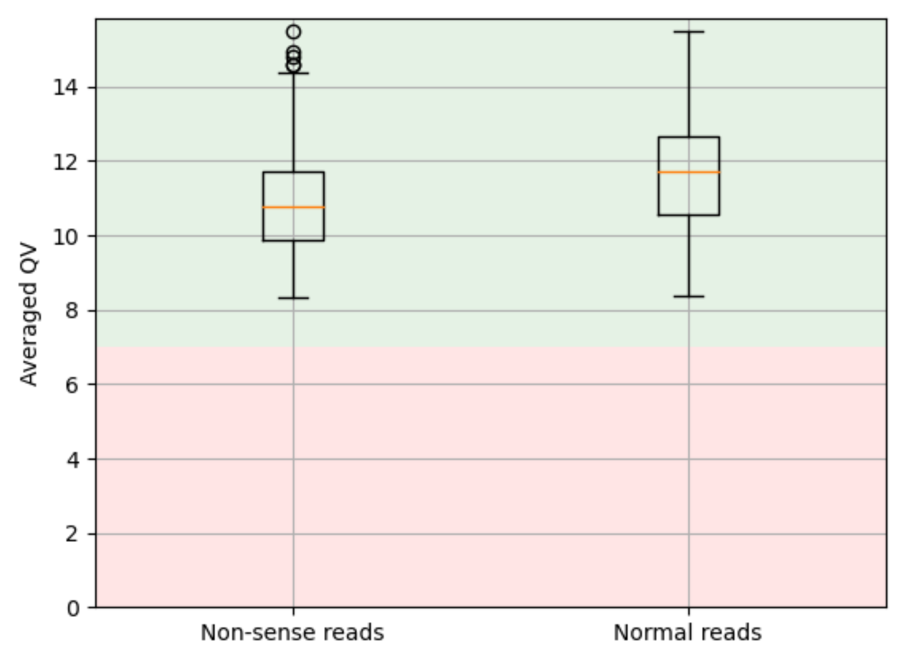

Number of sampled reads	5000
Mean per read coverage	18463.190
S.D. per read coverage	1959.305
Crude estimated Xome size	16559 (e = 18.0%)

GC contents

GC contents

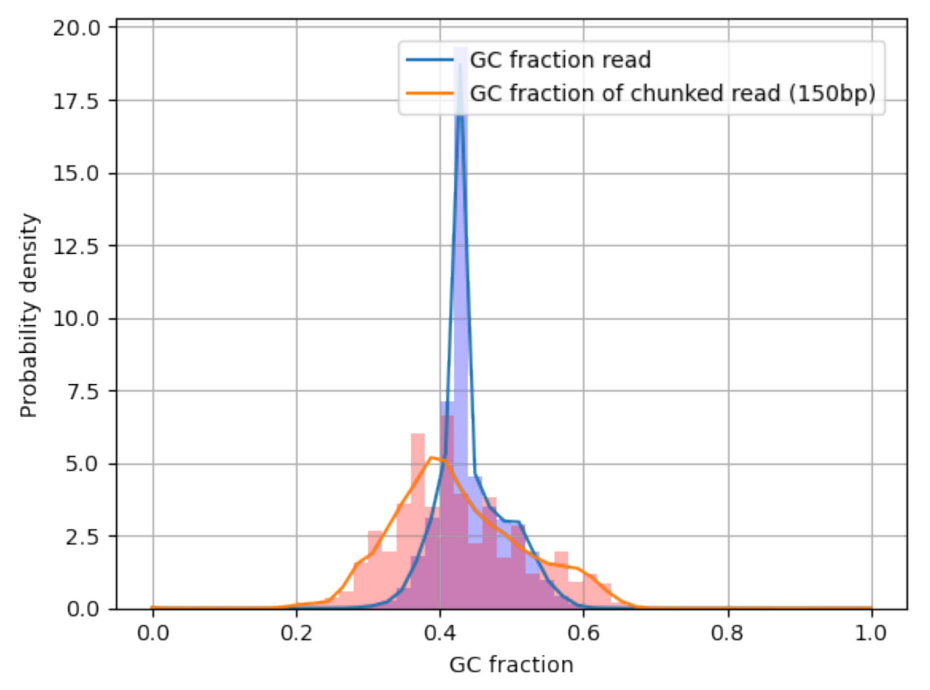

Mean per read GC content	44.004 %
s.d. per read GC content	4.516 %

Flanking region analysis

Flanking region analysis

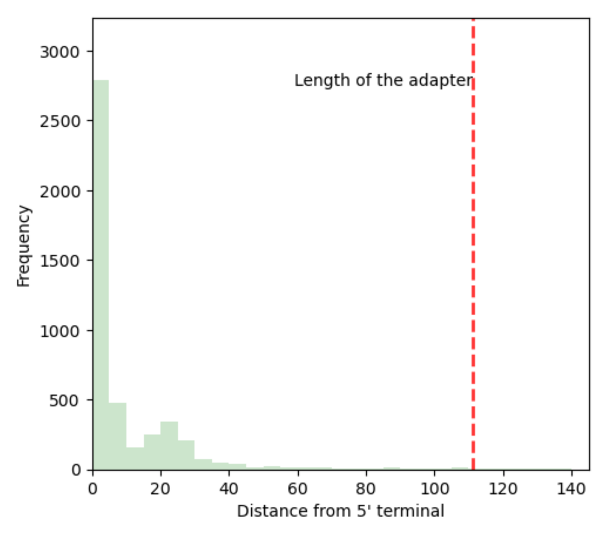

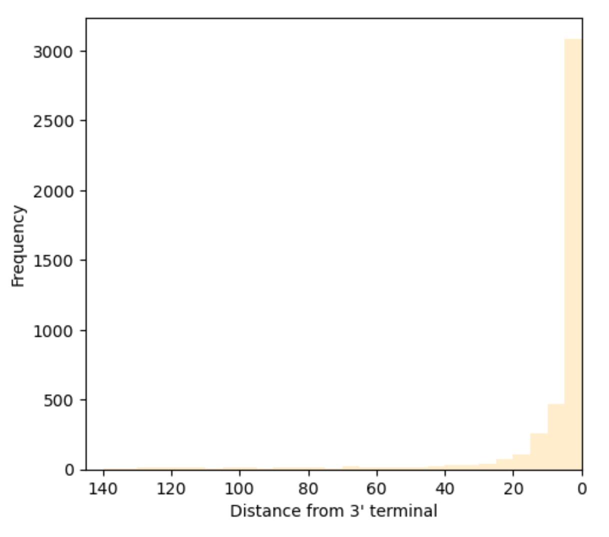

Sequence complexity

Sequence complexity

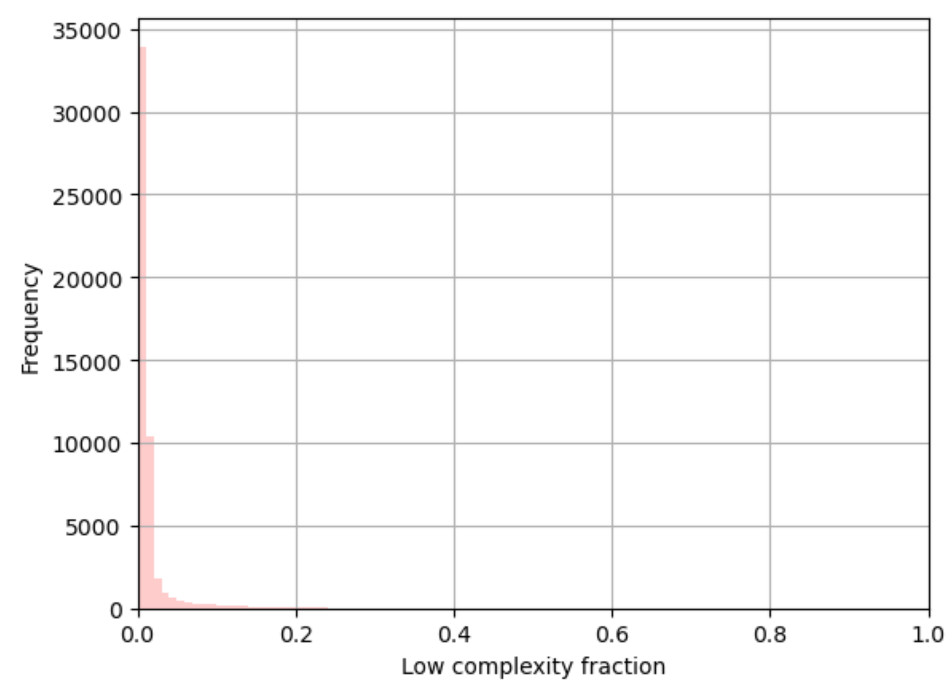

Supplement: Supplementary file 1 [file ijms-24-11163-s001.zip › File_S1.pdf]
